# Assessing the Impact of *Heyndrickxia coagulans* Administered Through Sugar-Free Chewing Gum on Dental Biofilm: A Double-Blind Randomized Controlled Trial

**DOI:** 10.3390/nu18060904

**Published:** 2026-03-12

**Authors:** Silvia Cirio, Giacomo Mantegazza, Claudia Salerno, Simone Guglielmetti, Aesha Allam, Guglielmo Campus, Maria Grazia Cagetti

**Affiliations:** 1 Department of Biomedical, Surgical and Dental Sciences, University of Milan, Via Beldiletto 1, 20142 Milan, Italy; claudia.salerno@ymail.com (C.S.); aesha.allam@unimi.it (A.A.); maria.cagetti@unimi.it (M.G.C.); 2 Department of Biotechnology and Biosciences, University of Milano-Bicocca, Piazza Della Scienza 2, 20126 Milan, Italy; giacomo.mantegazza@hest.ethz.ch (G.M.); simone.guglielmetti@unimib.it (S.G.); 3 Department of Cariology, Institute of Odontology, Sahlgrenska Academin, University of Gothenburg, 413 90 Gothenburg, Sweden; guglielmo.giuseppe.campus@gu.se; 4 Department of Oral and Maxillofacial Sciences, Sapienza University of Rome, 00161 Rome, Italy; 5 Department of Cariology, Saveetha, Dental College and Hospitals, SIMATS, Chennai 600077, Tamil Nadu, India

**Keywords:** *Heyndrickxia coagulans*, probiotic, dental plaque, oral microbiome, chewing gum, randomized clinical trial, metataxonomics

## Abstract

**Background:** *Heyndrickxia coagulans* has emerged as a candidate for oral health applications, and chewing gum offers a promising delivery method. This study evaluates whether *H. coagulans* delivered via sugar-free chewing gum can induce detectable changes in plaque microbial ecology. **Methods:** A randomized, double-blind, placebo-controlled clinical trial was conducted on 52 healthy adults. Participants consumed probiotic or control gum for 4 weeks. Dental plaque was collected at baseline (T_0_), mid-intervention (T_1_), end of intervention (T_2_), and one week post-intervention (T_3_). qPCR quantified *H. coagulans*, while 16S rRNA gene profiling assessed microbial diversity and taxonomic composition. Statistical analyses included rank-based difference-in-differences models, Wilcoxon and Mann–Whitney tests, and differential abundance inference based on negative binomial modeling. **Results:** Forty-four subjects completed the study. In the Intervention group, the strain was detected in 71.4% of participants at T_1_ and 61.9% at T_2_, and it persisted in 9.5% at T_3_. Differential abundance analysis revealed a broad depletion of taxa linked to oral dysbiosis at T_2_ with partial persistence at T_3_, along with selective enrichment of beneficial strains. **Conclusions:** *H. coagulans* delivered via chewing gum can reach the dental biofilm and induce modest, transient shifts in microbial composition. However, these biofilm ecology findings should be interpreted in the context of clinical outcomes.

## 1. Introduction

The human oral cavity hosts a diverse, site-specific microbial community, including bacteria, fungi, viruses, and archaea. Disruptions of this balanced ecosystem are linked to oral diseases such as caries and periodontitis [1,2].

Modulation of the oral microbiome through beneficial microorganisms, such as probiotics, has emerged as a promising adjunctive strategy to prevent or mitigate dysbiosis in the dental biofilm. Clinical and laboratory studies indicate that selected probiotic strains can antagonize oral pathogens, reduce inflammatory markers, and favorably alter microbial composition and cariogenic and periodontopathic bacteria, although reported effects vary across trials and depend on strain, dose, method of administration and study design [3,4,5].

Delivery modality critically determines the local exposure, retention time and ecological impact of probiotic organisms in the oral cavity. Chewing gum is an attractive delivery matrix for oral probiotics because it enables prolonged mechanical dispersion and salivary mixing, facilitates contact with tooth surfaces and interdental niches, and is associated with high patient acceptability and compliance [6]. Furthermore, advances in gum formulation and production allow inclusion of freeze-dried or microencapsulated cells while preserving viability and releasing kinetics. Consequently, chewing gum has been increasingly investigated as a targeted oral delivery system for bioactive agents, including probiotics [7,8,9].

*Heyndrickxia coagulans* (formerly *Bacillus coagulans*) is an endospore-forming lactic-acid-producing bacterium that combines industrial robustness with a favorable safety profile, features that have supported its use in a range of probiotic applications. Clinical evidence supports strain-specific benefits of *H. coagulans* for gastrointestinal benefits, and emerging preclinical and clinical data suggest that some strains can modulate microbial communities and host responses in ways potentially relevant to oral health. However, strain-level characterization, genomic safety screening, and context-specific evidence (particularly for oral delivery) remain important prerequisites for clinical translation [10,11,12]. In the oral cavity, the role of *H. coagulans* has been reported to antagonize the proliferation of *S. mutans*, reducing the incidence of caries [13,14,15,16,17]; moreover, there are some studies that have shown its beneficial effect in maintaining the health of the periodontium [18,19] and reducing the healing time of aphthous ulcers [20,21].

Despite growing interest in oral probiotics, evidence regarding their ability to reach dental plaque and induce measurable ecological changes within the biofilm remains limited. Few randomized controlled trials have evaluated strain-specific detection directly in plaque samples and assessed longitudinal microbiome responses [22].

Chewing gum represents a potentially advantageous delivery system for probiotics because it increases salivary flow, prolongs intraoral retention, and facilitates contact with dental surfaces. However, whether probiotic strains delivered via chewing gum can be detected in plaque and modulate its microbial ecology remains insufficiently explored.

The present randomized, double-blind, placebo-controlled trial was therefore designed to evaluate the ecological impact of *H. coagulans* SNZ1969^®^ on plaque microbiome composition.

## 2. Materials and Methods

### 2.1. Design of the Study

The present randomized controlled trial aimed to evaluate the ability of the probiotic *H. coagulans*, administered through sugar-free chewing gum, to colonize the dental plaque. The study was designed and conducted at the Department of Biomedical, Surgical and Dental Sciences, University of Milan (Milan, Italy), between September 2024 and April 2025, in accordance with the principles of the Declaration of Helsinki. Ethical approval was obtained from the Ethics Committee of the University of Milan (13 February 2024; protocol no. 24/24). Clinical trial registration: The study was initially conducted under confidentiality constraints due to the involvement of an industrial partner (Perfetti Van Melle S.p.a.). For this reason, certain aspects of the project were subject to confidentiality agreements during the development phase. Once these restrictions were lifted, we proceeded with the formal registration of the clinical trial in the ISRCTN Registry on 4 December 2025, after the study had already taken place (registration no. ISRCTN13055033).

### 2.2. Sample Selection

This study was conducted on healthy adult volunteers recruited from the staff of Perfetti Van Melle S.p.A. (PVM, Lainate, Italy) and from students enrolled in the undergraduate programs of dental hygiene and dentistry as well as the post-graduate specialty training program in pediatric dentistry at the University of Milan.

This study should be regarded as exploratory and hypothesis-generating, as no prior data were available to support a formal power calculation for plaque microbiome modulation. A sample size of 20 subjects per group, pragmatic and in line with a previous pilot microbiome trial [23], was chosen arbitrarily and later increased to 52 participants to account for potential dropouts [23]. The inclusion criteria were adult subjects aged 18 to 64 years, with at least 24 natural teeth (excluding third molars), gingival index and plaque index scores ≤ 2, and a stimulated salivary flow rate between 1.5 and 2.0 mL/min. Exclusion criteria included the presence of systemic diseases, pregnancy or lactation, history of drug abuse, smoking, use of fixed orthodontic appliances, and known allergies to any ingredients in the chewing gums used in the study. Brochures providing a concise overview of the study’s objectives and participation procedures were displayed near the lecture halls and in break rooms to recruit potential participants. A total of 56 individuals responded to the recruitment call. Two participants declined enrolment due to anticipated challenges in adhering to the study protocol. Subjects who gave their written consent to participate were interviewed to assess their eligibility based on the inclusion and exclusion criteria. They were then examined by a calibrated dentist (SC) to obtain their gingival index scores [24], gingival index [25] and stimulated salivary flow rate. Two participants were excluded due to potential allergies to chewing gum components. Finally, fifty-two eligible subjects were identified and enrolled. Participants were randomized into two groups (26 in the Intervention group and 26 in the Control group) using a computer-generated randomization system. Both participants and investigators were blinded to group allocation.

### 2.3. Chewing Gums Production

All chewing gums used in the study were produced and supplied by PVM. The sugar-free chewing gums (weight 2.1 g) were formulated with gum base (Gum Base Co., Lainate, Italy), food-grade polyols, excluding xylitol (proprietary blend; manufactured by Roquette Frères S.A. and Cargill Srl, Lestrem, France), food-grade intensive sweeteners (Ajinomoto Co., Inc., Tokyo, Japan), and flavors (Mondarom Selegroven AG, Bironico, Switzerland), and they incorporated the specific probiotic strain under investigation (*Heyndrickxia coagulans* SNZ1969^®^, provided by Sanzyme Biologics Ltd., Telangana, India).

The production process of chewing gum is summarized in the Supplementary Files (Supplementary File S2). Initially, the gum base is melted at 50 °C and sequentially combined with polyols, artificial intense sweeteners, and flavorings, achieving a homogeneous mixture. The freeze-dried probiotic biomass is incorporated as a final component below 50 °C to preserve spore integrity and viability. After mixing, in the rolling and scoring system, a mass of gum is extruded into a thick slab, which is then worked into a thinner and thinner foil by a series of rollers. Finally, the foil is shaped into single pieces by one or more cutting rollers. These pieces undergo cooling in a conditioning room before being panned, and afterward, individual pieces are prepared for packaging [26].

At the end of the production process, the amount of colony-forming units (CFUs) contained in the chewing gum was assessed according to the following protocol. The gum pieces in the bag were manually crushed and then processed in a Stomacher^®^ (Stomacher^®^ 3500 peristaltic homogenizer, Seward, West Sussex, UK) for 2 min. The resulting homogenized chewing gum mass was further serially diluted 1:10 in Maximum Recovery Diluent (MRD) buffer (GranuCult^®^ prime Peptone salt solution—Maximum recovery diluent, Merck KGaA, Darmstadt, Germany). Aliquots of 100 μL were plated in GYEA–agar (glycerol 5 g/L, yeast extract 2 g/L, K2HPO4 1 g/L, bromocresol green 10 mL/L from a stock of 5 g/L, agar 15 g/L pH 5.5, Merck KGaA, Darmstadt, Germany). Part of the samples underwent viable count after pasteurization (incubation in a water bath at 90 °C for 10 min) to quantify bacterial spores. The plates were incubated at 55 °C for 72 h in anaerobic conditions, established by incubating the plates in AnaeroJar Oxoid 2.5 L jars (Thermo Fisher Scientific^™^, Waltham, MA, USA) containing Anaerocult^™^ A (Merck KGaA, Darmstadt, Germany). The described procedures were repeated for each sample in triplicate. CFUs were identified by morphology and color and finally counted. The viable bacterial cell counts were expressed as colony-forming units per gram (CFU/g). At the end of the production process, the mean count of probiotics in one pellet of chewing gum was 5 × 108 CFUs.

The control chewing gum was matched to the test gum in terms of shape, color, and composition, and it was produced using the same process, but it did not contain any probiotics. It served not only to maintain participant blinding but also to account for potential mechanical effects of chewing, such as increased salivary flow and mechanical disruption of the biofilm.

Chewing gums were supplied in plain white containers coded as ‘green’ or ‘blue’ according to the group. The code was sealed by an independent monitor and not broken until the statistical analysis was finalized.

### 2.4. Use of Chewing Gum

The total study duration was seven weeks. All enrolled subjects received instructions for at-home oral hygiene and were provided with a manual toothbrush and a fluoride toothpaste (1450 ppm F) for use throughout the study period. Participants were instructed not to use any mouthwash or other oral hygiene products aside from those provided during the experimental period. Additionally, subjects were asked to refrain from using antibacterial or antibiotic medications (either topical or systemic), from taking probiotics, and from consuming chewing gum or other products containing xylitol. If the use of any of these products was necessary, participants were required to notify the investigators and would be subsequently excluded from the study.

The experimental period was structured as follows: an initial two-week washout phase, followed by a four-week intervention phase, and concluding with a one-week post-intervention phase. During the four-week intervention period, participants were instructed to consume the assigned chewing gum five times daily (after breakfast, mid-morning, after lunch, mid-afternoon, and in the evening after dinner), at least 30 min after brushing their teeth.

To encourage compliance and proper product intake, participants were provided with blister packs containing the exact number of chewing gums to be consumed between two consecutive follow-up visits (*n* = 70). They were asked to bring the empty blister pack at the subsequent visit to verify regular consumption. Additionally, participants were given a paper diary and instructed to record each chewing gum intake. Adherence to the protocol, at-home oral hygiene procedures, and any potential adverse effects were monitored using a custom-designed questionnaire, administered at each evaluation.

### 2.5. Outcomes Assessment

**Figure 1:**
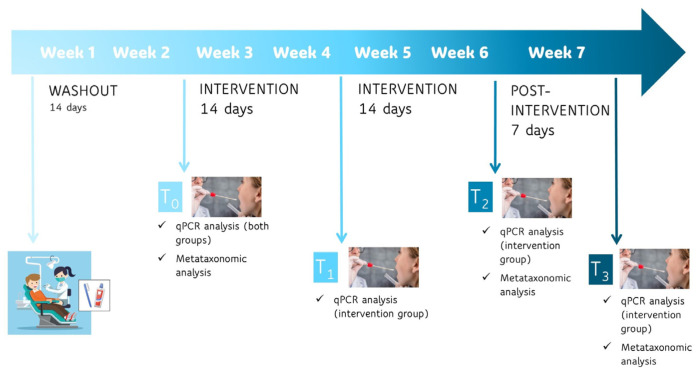
Chewing gum administration schedule and sample collection timeline.

Follow-up assessments were conducted at the following time points: after the initial two-week washout period (T_0_), after two weeks of chewing gum use (T_1_), after four weeks of chewing gum use (T_2_), and after an additional week at the end of the post-intervention period (T_3_) (Figure 1).

The primary outcome assessed was the characterization of the dental plaque microbial ecosystem. The secondary outcome was qPCR-based detection of *H. coagulans* SNZ1969^®^ in dental plaque in subjects who received the probiotic-containing chewing gum.

### 2.6. DNA Extraction from Dental Plaque Samples and qPCR Analyses

Dental plaque samples were collected from the buccal and lingual surfaces of all teeth using sterile swabs (FLOQSwabs^®^, Copan Italia S.p.A., Brescia, Italy). Dental plaque sampling was performed in the morning, at least 24 h after the last oral hygiene procedure and at least 2 h after food or drink intake, to standardize plaque accumulation conditions. Sampling was conducted before any clinical manipulation and prior to chewing gum use at each time point. The samples were then placed in tubes containing 1 mL of nucleic acid collection and preservation medium (eNAT^®^, Copan Italia S.p.A., Brescia, Italy). Samples were stored at 4 °C, transported to the laboratory within two hours from sampling, and immediately stored at −80 °C until analysis.

Total DNA was extracted from dental plaque samples using the QIAsymphony DSP Virus/Pathogen Midi Kit^®^ (Qiagen, Milan, Italy), following the manufacturer’s instructions. DNA concentration was determined fluorometrically with a Qubit 4 Fluorometer^®^ (Thermo Fisher Scientific, Segrate, Italy), and 10 ng of template DNA from each sample was used per real-time quantitative PCR (qPCR) to detect and quantify *H. coagulans* SNZ1969^®^ cells. Strain-specific quantification was performed as described in Perotti et al. [27], with minor modification on amplification condition. Thermal cycling conditions were 98 °C for 30 s, followed by 40 cycles of 96 °C for 2 s and 59 °C for 5 s. Fluorescence was recorded at the end of each 59 °C step, and amplification specificity was verified by post-run melt-curve analysis. Cell-equivalent counts were calculated from a standard curve generated with DNA extracted from a pure culture of the target strain. Under the detection limit (*u.d.l*.) has been determined at <1.3 Log_10_ cells/ng.

### 2.7. Metataxonomic Analysis via 16S rRNA Gene Profiling

DNA extracted from dental plaque, as described above, was subjected to 16S rRNA gene amplicon sequencing targeting the V3–V4 hypervariable region using primers 515F and 806R. Sequencing was performed on an AVITI platform (Element Biosciences, San Diego, CA, USA) using paired-end 2 × 300 bp chemistry at the Center for Omics Sciences (COSR), San Raffaele Hospital (Milan, Italy). The sequencing provider supplied raw paired-end reads in FASTQ format together with run-level quality reports (FastQC) and a technical report. Sequence data were processed using QIIME 2 (version 2024.5). After demultiplexing, reads were quality filtered, denoised, merged, and checked for chimeras using the DADA2 plugin (denoise-paired), generating an amplicon sequence variant (ASV) feature table and representative sequences. Trimming and truncation parameters were selected based on inspection of per-base quality score profiles. α- and β-diversity analyses were performed on the ASV table using the q2-diversity plugin. α-diversity metrics included observed ASVs, Shannon entropy, Faith’s phylogenetic diversity, and Pielou’s evenness. Beta-diversity was assessed using Bray–Curtis, Jaccard, weighted UniFrac, and unweighted UniFrac distance metrics. Taxonomic classification of ASVs was carried out using a Naive Bayes classifier trained on the SILVA reference database (release 138/138.1, 99% OTUs), using reference sequences trimmed to the 515F–806R region. Feature tables were collapsed to higher taxonomic ranks (from phylum to genus) when required for downstream statistical analyses (Supplementary File S3).

### 2.8. Statistical Analysis

To assess the Time × Treatment effect on α-diversity indices, a rank-based “difference-in-differences” strategy was used:

1. For each subject and metric, paired deltas were computed (T_2_−T_0_, T_3_−T_0_, and T_3_−T_2_).
2. Delta distributions were compared between the Probiotic and Control groups using two-sided Mann–Whitney U (MWU) tests, the non-parametric analogue of an interaction test when time is expressed as paired differences.
3. Within-group temporal changes were assessed with two-sided Wilcoxon signed-rank tests on raw values for each group (Control, Probiotic) and time pair.
4. For MWU, effect sizes were reported as rank-biserial correlation (r); median deltas were also reported for both groups.
5. Multiple testing across contrasts was controlled using the Benjamini–Hochberg false discovery rate (FDR; q-values).

Analyses were performed in Python 3.13 (SciPy/NumPy/Pandas; Matplotlib for visualization). For β-diversity metrics, the first two principal coordinates (PC1 and PC2) were extracted and displayed in scatter plots stratified by treatment (Control vs. Probiotic) and time point (T_0_, T_2_, T_3_). Temporal trajectories for each subject were visualized as arrows connecting time points in chronological order (T_0_ → T_2_ → T_3_). Statistical differences across time points were assessed using two complementary approaches: (i) pairwise Mann–Whitney U tests for PC1 and PC2 between T_0_ vs. T_2_, T_1_ vs. T_3_, and T_2_ vs. T_3_ within each group; and (ii) Analysis of Similarities (ANOSIM, 999 permutations) on Euclidean distance matrices derived from PC1 and PC2, both for global comparisons (T_0_, T_2_, T_3_) and pairwise contrasts.

Differential abundance analysis was performed on the ASV count table using a generalized linear model with negative binomial distribution, implemented in a DESeq2-like framework. The model included time, treatment, and their interaction (time × treatment) as fixed effects, while subject was included as a blocking factor to account for the longitudinal study design and repeated measures. Size factor normalization was performed using the median-of-ratios method, and statistical significance was assessed using Wald tests. To reduce model instability due to sparse features, taxa with low prevalence (present in fewer than X% of samples or with total counts below Y across the dataset) were filtered prior to model fitting. *p*-values were adjusted for multiple testing using the Benjamini–Hochberg false discovery rate (FDR) procedure. Given the compositional nature of 16S rRNA gene sequencing data, differential abundance results were interpreted with caution. As a sensitivity analysis, an alternative compositional-aware method was applied (e.g., ANCOM-BC/ALDEx2/MaAsLin2), and concordance in the direction of effects for key taxa was evaluated.

## 3. Results

### 3.1. Sample Characteristics

**Figure 2:**
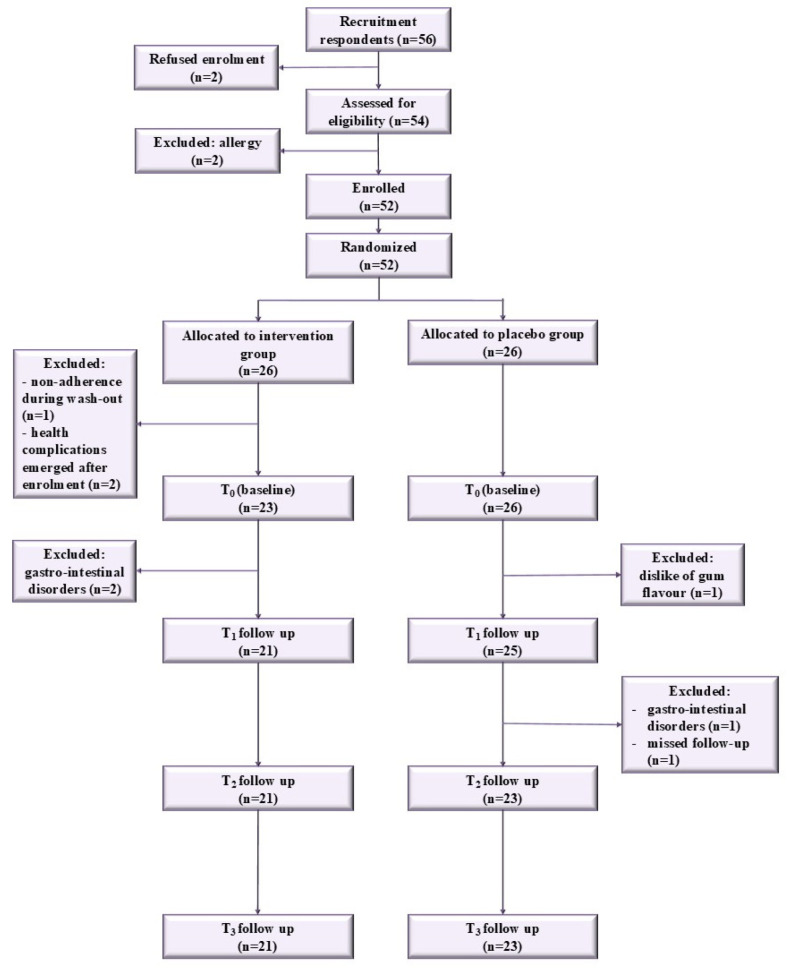
Flow diagram of participant recruitment, randomization, and follow-up in the clinical trial.

A total of 52 volunteers (26 per group) started the 2-week washout period. Three participants in the Intervention group were excluded: one for non-compliance with the washout instructions, and two owing to health complications that emerged after enrolment. Subsequently, chewing gum administration was initiated by 23 participants in the Intervention group and 26 in the Control group. During the study, one participant in the Control group dropped out due to dislike of the gum’s taste, while another participant in the Intervention group withdrew because of gastrointestinal disorders; therefore, they were excluded from the first follow-up (T_1_). Before the second follow-up (T_2_), two additional participants in the Control group were excluded: one discontinued chewing gum due to gastrointestinal disorders, and another missed the follow-up for personal reasons. Ultimately, 21 participants in the Intervention group and 23 in the Control group completed the study (drop out 13.5%). Figure 2 and Supplementary File S4 show recruitment, randomization, and follow-up of participants in the clinical trial.

The mean age of participants was 27.9 years (29.3 ± 10.5 in the Intervention group and 27.0 ± 8.2 in the Control group), and 85.7% were female (20 in the Intervention group and 22 in the Control group) (Table 1).

A total of 11 participants (7 in the Intervention group and 4 in the Control group) did not fully adhere to the chewing gum regimen; the mean of missed gums was 1.0 ± 2.4/140 (range 0–12). Nine participants (5 in the Intervention group and 4 in the Control group) reported disliking the taste, texture, or size of the chewing gum. Furthermore, 17 participants (10 in the Intervention group and 7 in the Control group) reported gastrointestinal side effects, including bloating, reflux, gastritis, and abdominal pain. No statistically significant differences were found between groups (Table 1).

Final analyses were conducted on the 44 subjects who completed the study (mean age: 29.5 in the Intervention group and 27.4 in the Control group). No statistically significant differences were observed in age or sex between participants who used the probiotic chewing gum and those who used the control chewing gum.

### 3.2. Presence of H. coagulans in Dental Plaque Samples

**Figure 3:**
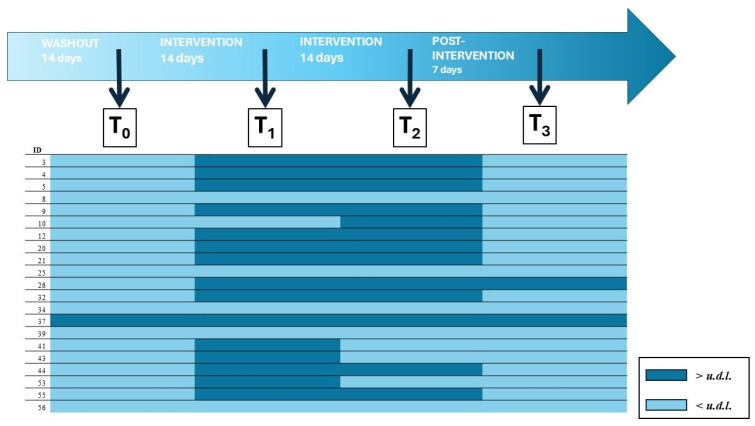
qPCR-based detection levels of probiotic strain *H. coagulans* at different follow-up points of subjects in Intervention group. *u.d.l*., under detection limit (<1.3 Log_10_ cells/ng).

A total of 107 dental plaque samples (T_0_ *n* = 44; T_1_ *n* = 21; T_2_ *n* = 21; T_3_ *n* = 21) were analyzed by qPCR for the detection of strain *H. coagulans* SNZ1969. Values are expressed as Log_10_ cell equivalents per ng of DNA (Log_10_ cells/ng). The limit of detection (LOD) was 1.3 Log_10_ cells/ng; values below the LOD are reported as under the detection limit (*u.d.l*.). In the Control group (*n* = 23), no sample was positive at T_0_ (0/23). In the Intervention group (*n* = 21), one volunteer was weakly positive at T_0_ (1/21; 4.8%). During the Intervention, positivity was observed in 71.4% of subjects at T_1_ (15/21) and 61.9% at T_2_ (13/21); overall, 16/21 (76.2%) were positive at least once across T_1_–T_2_, and 12/21 (57.1%) at both time points. One week after discontinuation (T_3_), positivity persisted in 2/21 subjects (9.5%; subject ID 26 and 37), while the others were *u.d.l.* All five non-responders (subjects coded as number 8, 25, 34, 39, 56) remained *u.d.l*. at T_1_–T_3_. Among positives, the target load showed median values of 2.3 Log_10_ cells/ng (IQR 2.0–2.6; range 1.4–4.8) at T1 and 2.4 (2.0–2.9; 1.6–5.1) at T_2_. At T_3_, the two subjects who were still positive exhibited high loads (median 4.4; range 4.2–4.7 Log_10_ cells/ng). The most marked individual dynamics were observed in subjects 32 (1.4→4.8 Log_10_ cells/ng from T_1_ to T_2_) and 37 (1.6 → 4.8 → 5.1 → 4.2 from T_0_ to T_3_), whereas 26 increased from 2.0 to 4.2 and then 4.7 across T_1_, T_2_, and T_3_, respectively. Analytical specificity is supported by the absence of signal in controls and concordant melting curves. The results described above are reported in Table 2 and Figure 3.

### 3.3. Analysis of the Bacterial Community Structure of Dental Plaque: α-Diversity

**Figure 4:**
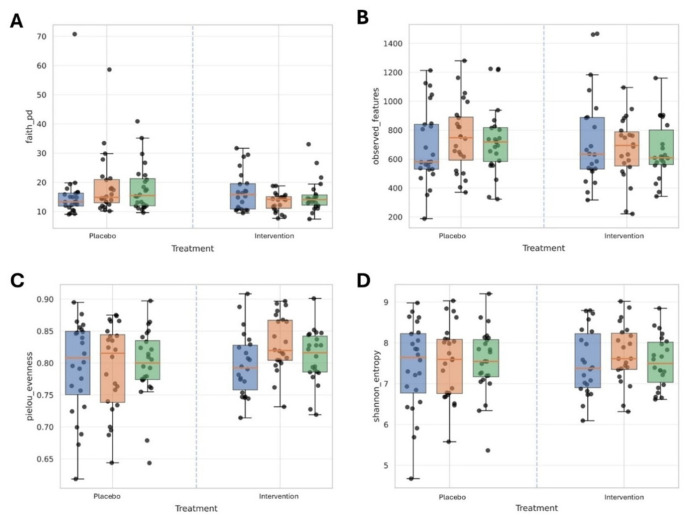
Alpha-diversity metrics of dental plaque microbiota across study groups and time points. Boxplots represent the distribution of (**A**) Faith’s phylogenetic diversity, (**B**) observed features (richness), (**C**) Pielou’s evenness, and (**D**) Shannon entropy in the Control and Intervention groups at baseline (T_0_), end of intervention (T_2_), and follow-up (T_3_). Each dot corresponds to an individual sample. Significant differences in deltas between groups were observed during the Intervention phase (T_0_–T_2_) for Faith’s phylogenetic diversity (*p* = 0.0027) and Pielou’s evenness (*p* = 0.0160), with the Intervention group showing increased evenness (*p* = 0.0103, Wilcoxon test). No significant group-dependent differences were detected for observed features or Shannon entropy.

In the longitudinal analysis of dental plaque α-diversity metrics, the Time × Treatment interaction, evaluated non-parametrically (Mann–Whitney U on deltas), showed a significant divergence between Intervention and Control groups for Faith’s phylogenetic diversity (*p* = 0.0027; *n*_control = 24, *n*_probiotic = 22; r = −0.52) during the Intervention (T_0_ → T_2_): the median Δ increased in the Control group (+1.68) and decreased in the Intervention group (−2.48). For Pielou’s evenness, a between-group difference was observed (*p* = 0.0160; r = +0.42), with median Δ ≈ −0.0026 in the Control group and +0.0367 in the Intervention group; accordingly, evenness increased within the Intervention group (Wilcoxon *p* = 0.0103) but not within the Control group (*p* = 0.944). Faith’s PD also changed within both groups over T_0_→T_2_ (Control *p* = 0.0340; Intervention *p* = 0.0275), albeit in opposite directions. No treatment-dependent differences emerged for observed features or Shannon entropy, nor in the T_0_ → T_3_ or T_2_ → T_3_ contrasts for any metric. After FDR correction across all tests, however, no result reached q < 0.005 (minimum q = 0.0967 for Faith T_0_ → T_2_); the signals observed over T_0_ → T_2_ should therefore be interpreted as exploratory/hypothesis-generating (Table 3 and Figure 4).

### 3.4. Analysis of the Bacterial Community Structure of Dental Plaque: β-Diversity

**Figure 5:**
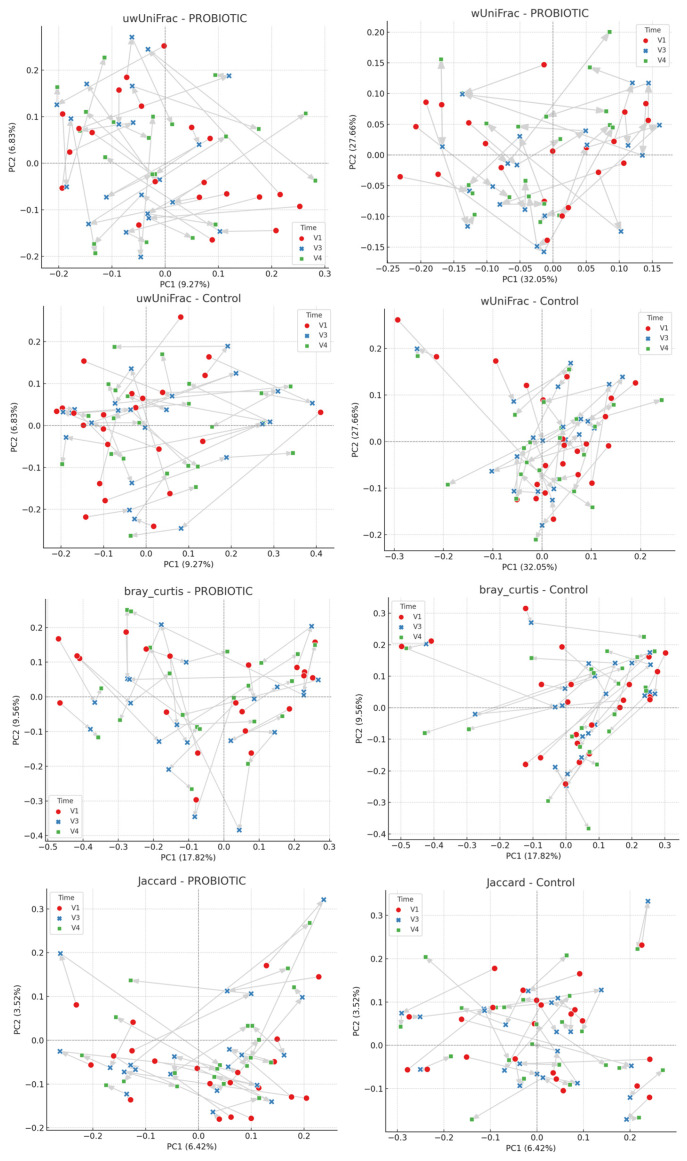
Principal coordinate analysis (PCoA) plots based on weighted UniFrac (wUniFrac), unweighted UniFrac (uwUniFrac), Jaccard, and Bray–Curtis dissimilarities. The first two coordinates (PC1 and PC2) are shown, with the proportion of variance explained indicated on each axis. Samples are colored by time point (red = T_0_, blue = T_2_, green = T_3_) and stratified by treatment group (Control and Intervention). Arrows connect longitudinal samples from the same subject (T_0_ → T_2_ → T_3_), illustrating temporal trajectories. No clear clustering by time point was observed across either treatment group or beta-diversity metric.

Ordination plots showed substantial overlap among time points for both treatment groups and across all four β-diversity metrics, with no clear clustering by time. Mann–Whitney U tests did not reveal significant differences between time points in either the Control or Intervention groups (all *p* > 0.05). Some borderline trends were observed, including unweighted UniFrac in the Control group (T_0_ vs. T_3_, PC1, *p* ≈ 0.075), unweighted UniFrac in the Intervention group (T_0_ vs. T_2_, PC1, *p* ≈ 0.125), and Bray–Curtis in the Intervention group (T_0_ vs. T_2_, PC2, *p* ≈ 0.107), but none reached significance. ANOSIM confirmed these findings: global comparisons yielded R values close to zero (wUniFrac R ≈ −0.027, *p* = 0.115; uwUniFrac R ≈ −0.012, *p* = 0.593; Jaccard R ≈ −0.036, *p* = 0.050; Bray–Curtis R ≈ −0.031, *p* = 0.075 in the Control group), indicating negligible group separation. Pairwise ANOSIM tests produced similarly low R values, with *p*-values > 0.05 in all cases (Table 4 and Table 5 and Figure 5).

### 3.5. Analysis of the Bacterial Community Structure of Dental Plaque: Bacterial Taxa

Using a DESeq2-like model with a Time × Treatment interaction, probiotic-associated shifts in dental plaque were identified, being most pronounced at the end of supplementation (T_2_) and, more selectively, persisting at follow-up (T_3_), thus highlighting taxa with divergent temporal trajectories between groups. At T_2_, the Intervention group exhibited significant reductions across multiple lineages, including *Actinobacteriota* (orders *Micrococcales*, *Propionibacteriales*), *Bacteroidota* (orders *Flavobacteriales* and *Sphingobacteriales*, as well as *Bacteroidales* members such as *Rikenellaceae*_RC9_gut_group), *Bacillota* (orders *Lactobacillales*, *Staphylococcales*), *Patescibacteria* (order *Saccharimonadales*), and *Pseudomonadota* (orders *Burkholderiales*, *Pasteurellales*, *Pseudomonadales*). At lower ranks, decreases encompassed the families *Micrococcaceae* (genus *Rothia*), *Carnobacteriaceae*, *Streptococcaceae*, *Staphylococcaceae*, *Gemellaceae*, *Weeksellaceae* (genus *Bergeyella*), *Lentimicrobiaceae* (genus *Lentimicrobium*), *Burkholderiaceae* (genus *Lautropia*), *Neisseriaceae* (genus *Neisseria*), *Pasteurellaceae*, and *Moraxellaceae*. In contrast, the genus *Lachnoanaerobaculum* (member of the family *Lachnospiraceae*) increased in the Intervention group. At T_3_, changes remained but were more selective, with sustained decreases in clinically relevant taxa including the genera *Actinomyces* (family *Actinomycetaceae*), *Prevotella* and *Alloprevotella* (*Prevotellaceae)*, *Rikenellaceae*_RC9_gut_group, *Tannerella* (*Tannerellaceae*), *Gemella* (*Gemellaceae*), Leptotrichia (*Leptotrichiaceae*), *Kingella* (*Neisseriaceae*), *Moraxella* (*Moraxellaceae*), and *Treponema* (*Spirochaetaceae*), alongside reductions at higher ranks (families *Lachnospiraceae*, *Neisseriaceae*, *Pseudomonadales*, *Saccharimonadaceae*). Concomitantly, an unclassified *Actinomycetaceae* genus (F0332) and *Selenomonadaceae* increased (Table 6).

Overall, probiotic supplementation was associated with a decrease in the relative abundance of numerous oral taxa (with maximal effects at T_2_ and still evident at T_3_) and a selective enrichment of specific commensals or low-abundance taxa.

## 4. Discussion

The aggregate qPCR data indicates that the administration of *H. coagulans* SNZ1969^®^ via chewing gum led, in most subjects, to detectable levels of the bacterial DNA in dental plaque within 2 weeks, with positivity maintained in ~62% at 4 weeks and persisting in ~10% one week after discontinuation. The absence of signals at baseline (T_0_) in the Control group and in 95% of subjects in the Intervention group suggests that positivity was attributable to the Intervention rather than pre-existing presence of the target bacterium. In a previous study, conducted with the same strain (*H. coagulans* SNZ1969^®^) delivered through the identical chewing gum formulation, persistence in saliva was assessed by plating and cultivation, and the strain was shown to remain detectable for at least 2 h post-administration [28]. Median loads during the intervention were ~2.3–2.4 Log_10_ cells/ng, with peaks exceeding 4.7–5.1, and individual trajectories (subject with ID 26, 32, 37) were consistent with genuine colonization or local accumulation of cells/spores in subsets of “responders”. By contrast, 5/21 subjects remained consistently negative, pointing to inter-individual variability (e.g., adherence, oral physiology, dental plaque microecology) that warrants further investigation. However, based on the available data, it was not possible to determine whether probiotic administration exerted ecological or functional effects on the oral microbiome in subjects in whom *H. coagulans* DNA remained undetectable. Probiotic activity does not necessarily rely on stable colonization of the target niche and may instead occur through transient ecological interactions, metabolite-mediated signaling, or modulation of host responses. In the present study, the relatively small sample size and the reliance on detection of strain-specific DNA likely limited the ability to capture such subtle or indirect microbiome effects [29,30]. Normalization per ng of total DNA reduced, but did not eliminate, variability due to sampling. Nonetheless, such inter-individual heterogeneity was also noted in previous oral probiotic clinical studies [28,31]. It should be emphasized, however, that qPCR detects target DNA without distinguishing between live or metabolically active cells, spores, or free DNA; the term “colonization” should therefore be used cautiously in the absence of viability or metabolic evidence. Notably, *H. coagulans* itself did not emerge as a dominant taxon in the amplicon-based profiling, a finding that is consistent with the limited sensitivity and taxonomic resolution of 16S rRNA gene sequencing for low-abundance, spore-forming, non-resident taxa [32]. Regarding α-diversity, during active administration (T_0_ → T_3_), the Intervention group exhibited distinct ecological trajectories from the Control group. Specifically, (i) Faith’s PD decreased in the Intervention group while increasing in the Control group, and (ii) evenness increased in the Intervention group with minimal change in the Control group. The combination of reduced phylogenetic breadth and increased evenness suggests selective reshaping of community structure, limiting phylogenetic diversity while redistributing relative abundances more uniformly, as observed in other oral microbiome modulation studies [33,34]. Such a pattern is consistent with targeted competitive interactions and/or niche replacement, reducing dominance effects within the community. Importantly, no effects persisted to T_3_ (post-cessation), indicating reversibility and dependence on continued exposure. However, although T_0_→T_2_ differences reached nominal significance (*p* < 0.05), they did not withstand FDR correction, underscoring the need for cautious interpretation and validation in larger, adequately powered cohorts. Overall, these findings suggest that probiotics delivered via chewing gum can transiently modulate dental plaque’s ecology, increasing evenness while altering phylogenetic composition, without durable effects after discontinuation. The functional and clinical implications of this diversity signature (lower Faith’s PD with higher evenness) warrant further study, ideally linking taxonomic and functional profiles to oral health outcomes. For β-diversity, no significant temporal changes were detected across any of the four metrics in either group. Both Mann–Whitney tests and ANOSIM consistently indicated no robust differences among time points (R statistics close to zero; non-significant *p*-values). Borderline trends (e.g., Jaccard and Bray–Curtis in 17 controls) were weak and likely reflect random variation rather than systematic changes. Thus, within the studied timeframe, the Intervention did not produce measurable shifts in dental plaque microbial structure at the β-diversity level, a pattern consistent with other RCTs showing stability in overall community structure despite transient probiotic effects [5,31]. This apparent stability of overall community structure despite taxon-level modulation suggests that probiotic supplementation may act primarily through fine-scale compositional tuning rather than large-scale community restructuring. The ‘DESeq2-like’ differential analysis with a Time × Treatment interaction (subject included as a blocking factor) revealed divergent compositional trajectories between the Intervention and Control groups, with broad, acute suppression at T_2_ (end of dosing) affecting orders/families typically associated with dysbiotic states, as well as a more selective persistence at T_3_ (follow-up), featuring maintained decreases in lineages linked to periodontal disease and limited enrichments of potential commensals. Specifically, within the phylum *Pseudomonadota* (formerly Proteobacteria), at T_2_, coordinated reductions in *Burkholderiales* (including *Burkholderiaceae* and *Lautropia*), *Pasteurellales*/*Pasteurellaceae*, and *Pseudomonadales*/*Moraxellaceae* were observed; at T_3_, these were joined by decreases in *Neisseriaceae* (including *Neisseria* and *Kingella*) and in *Pseudomonadales* (with *Moraxella*). These groups include oral/respiratory opportunists and inflammation-associated colonizers, and their attenuation is consistent with competitive exclusion, resource depletion, and/or microenvironmental remodeling induced by supplementation [30,34,35]. Furthermore, it should be considered that Neisseria and Kingella, together with other genera such as *Rothia*, are key nitrate reducers. Therefore, it can be speculated that their decline may lower the overall capacity for nitrate → nitrite → NO conversion, with potential functional consequences. To determine whether this ecological shift is beneficial or entails a detrimental loss of function, microbiological analyses should be complemented by functional assessments, including salivary nitrate/nitrite levels and exhaled NO [36,37,38]. These functional interpretations remain hypothesis-generating and require direct metabolomic or transcriptomic validation. Within the phylum *Bacteroidota* (formerly *Bacteroidetes*), at T_2_, we found decreases in *Flavobacteriales* (*Flavobacteriaceae*, *Weeksellaceae*/*Bergeyella*) and *Capnocytophaga*, and at T_3_, marked decreases in *Prevotella*/*Alloprevotella*, *Tannerella*, and *Rikenellaceae*_RC9. These genera/lineages are consistently associated with gingivitis and periodontitis, and the temporal comparison between the Intervention and the Control groups suggests that the probiotic either reduced the increases occurring in the Control group or reinforced the decreases, indicating a shift away from mature proteolytic anaerobic consortia [38,39,40,41]. In the phylum *Bacillota* (formerly *Firmicutes*), at T_2_, reductions in *Staphylococcales* (including *Gemellaceae*) and *Lactobacillales* (*Carnobacteriaceae*, *Streptococcaceae*) were noted, whereas at T_3_ *Gemella* and the family *Lachnospiraceae* declined; in the opposite direction, *Lachnoanaerobaculum* increased at T_2_, consistent with the saccharolytic/acidogenic nature of the genus and with possible transient niches opened by treatment, followed by family-level re-equilibration at T_3_ [42,43]. Among anaerobic “late colonizers” and “bridge” organisms, sustained T_3_ decreases were recorded for *Leptotrichia* (*Fusobacteriota*) and *Treponema* (*Spirochaetota*), in line with an attenuation of mature/inflammatory consortia after supplementation [43,44,45]. In the phylum *Actinobacteriota* (formerly *Actinobacteria*), T_2_ showed declines in *Micrococcales*/*Micrococcaceae* (*Rothia*) and *Propionibacteriales* (typical early colonizers of hard surfaces), while at T_3_ *Actinomyces* decreased and an unclassified *Actinomycetaceae* (F0332) increased, indicating taxon-specific selection rather than homogeneous phylum-level shifts. Notably, *Rothia* is a known nitrate reducer, with 18 functional implications analogous to the note above on Neisseriaceae [46,47,48]. For candidate phyla radiation (*Patescibacteria*/TM7), *Saccharimonadaceae* (including *Candidatus Saccharimonas*) showed consistently negative coefficients at T_2_ and T_3_. Since TM7 interacts epibiotically/parasitically with *Actinomyces* and is implicated in periodontal inflammation, its sustained reduction is indicative of a potentially favorable microbial rebalancing. Nevertheless, caution is warranted because large apparent changes in low-abundance taxa can be strongly influenced by data sparsity [49,50]. Overall, the broad-pattern suppression at T_2_ followed by selective persistence at T_3_ is consistent with targeted ecological remodeling of the bacterial biofilm, with depletion of proteolytic/anaerobic consortia (e.g., *Prevotella*/*Tannerella*/*Treponema* and various *Pseudomonadota*) and circumscribed rebounds of commensals (e.g., *Lachnoanaerobaculum* at T_2_; *Actinomycetaceae* F0332 and *Selenomonadaceae* at T_3_). This type of “targeted and modest-magnitude” effect aligns with clinical evidence on the use of probiotics as adjuncts in periodontal therapy [51,52].

The marked inter-individual variability that was observed suggests that host factors, baseline microbiome composition, and ecological resilience may influence responsiveness to probiotic supplementation. Moreover, the disappearance of most signals after cessation indicates that continued administration may be necessary to sustain ecological effects. It should also be acknowledged that chewing gum itself may influence oral ecology through increased salivary flow and mechanical plaque disruption. Although the placebo-controlled design accounts for these factors, subtle mechanical effects may have contributed to microbiome variability in both groups.

Methodologically, the log-linear approach mirrors DESeq2 in applying size-factor normalization and count-based inference, but it uses heteroskedasticity-robust standard errors to estimate the Time × Treatment interaction. However, the compositional structure of 16 subjects’ data, together with potential heterogeneity within families or genera (for example, *Selenomonadaceae* increasing while *Selenomonas* decreases), highlights important limitations. These considerations argue for focusing on q-values and effect directions, taking taxon prevalence into account, and planning follow-up analyses at the strain level. In addition, shotgun metagenomics would be needed to achieve functional resolution. Within these constraints, interpreting the results in a difference-in-differences framework remains consistent with best practice for longitudinal repeated-measures microbiome studies [52,53,54,55,56].

Importantly, this study did not include clinical oral health endpoints such as caries incidence, gingival inflammation, or periodontal parameters. Therefore, the observed microbiome changes should be interpreted as ecological shifts within the dental biofilm rather than evidence of clinical benefit. Whether such compositional modulation translates into improved oral health outcomes remains to be determined.

## 5. Conclusions

In this randomized, placebo-controlled study, qPCR experiments showed that *H. coagulans* SNZ1969^®^ delivered via chewing gum reached detectable levels in most treated volunteers during dosing (71.4% at 2 weeks; 61.9% at 4 weeks), with persistence in 9.5% one week after discontinuation. Median loads were ~2.3–2.4 Log_10_ cells/ng during the intervention, and peak values exceeded 5 Log_10_ cells/ng in responders, whereas all control samples remained negative. These findings support effective release and local accumulation, although viability could not be confirmed and inter-individual variability was substantial. Metataxonomic analyses indicated a transient reshaping of α-diversity during the intervention (decrease in Faith’s PD with a concomitant increase in evenness in the Intervention group), no effects surviving FDR correction, and no significant time-dependent separation at the β-diversity level. Difference-in-differences inference on taxa revealed broad depletion at the end of probiotic administration (T_2_), with selective persistence at follow-up (T_3_), in lineages frequently implicated in periodontitis (e.g., *Prevotella*/*Alloprevotella*, *Tannerella*, *Treponema*, *Moraxellaceae*/*Moraxella*, *Neisseriaceae*/*Neisseria*/*Kingella,* and *Leptotrichia*), alongside limited enrichments of putative commensals (e.g., *Lachnoanaerobaculum* at T_2_; *Actinomycetaceae* F0332 and *Selenomonadaceae* at T_3_).

Overall, the data suggest that *H. coagulans* SNZ1969^®^ administered through chewing gum may induce modest and transient ecological shifts in dental plaque composition. These effects were reversible after discontinuation and should be interpreted cautiously given the exploratory design and limited sample size. Confirmation in larger, adequately powered studies incorporating viability assays, functional analyses, and clinical endpoints is needed before drawing definitive conclusions.

## Figures and Tables

**Table 1:** Characteristics of participants, compliance and adverse effects reported in the two study groups during the experimental period.

	Intervention	Control	Total	*p* Value
	*N =* 23	*N* = 26	*N* = 49	
Age (years)				
Mean (SD)	29.3 (10.5)	27.0 (8.2)	27.9 (9.0)	0.665 ^c^
Range	20.0; 55.0	20.0; 53.0	20.0; 55.0	
Sex (*n* (%))				
F	20 (87.0)	22 (84.6)	42 (85.7)	1.000 ^a^
M	3 (13.0)	4 (15.4)	7 (14.3)	
Compliance				
*N* (%) of subjects that skipped chewing gum during Intervention period	7 (30.4)	4 (15.4)	11 (22.5)	0.306 ^a^
Chewing gum skipped				
Mean (SD)	1.3 (2.8)	0.8 (2.1)	1.0 (2.4)	0.285 ^c^
Range	0.0; 12.0	0.0; 8.0	0.0–12.0	
Dislike (*n* (%) of subjects that disliked chewing gum)	5 (21.7)	4 (15.4)	9 (18.4)	0.716 ^a^
Adverse effect (*n* (%) of subjects that referred to gastrointestinal disorders)	10 (43.5)	7 (26.9)	17 (34.7)	0.224 ^b^

*N*: number; SD: standard deviation; M: male; F: female. Normality and heteroskedasticity of continuous data were assessed with Shapiro–Wilk test. ^a^—Fisher’s exact test; ^b^—Chi-square test; ^c^—Mann–Whitney U test.

**Table 2:** qPCR-based detection of probiotic strain of *H. coagulans* in oral samples from subjects in Intervention group. Values are reported as Log_10_ of target-gene copies (cell equivalents) per ng of extracted DNA (Log_10_ cells/ng).

ID	T_0_	T_1_	T_2_	T_3_
3	*u.d.l.*	2.88	2.68	*u.d.l.*
4	*u.d.l.*	2.44	2.03	*u.d.l.*
5	*u.d.l.*	2.27	1.63	*u.d.l.*
8	*u.d.l.*	u.d.l.	*u.d.l.*	*u.d.l.*
9	*u.d.l.*	2.73	2.94	*u.d.l.*
10	*u.d.l.*	u.d.l.	1.67	*u.d.l.*
12	*u.d.l.*	2.21	2.25	*u.d.l.*
20	*u.d.l.*	2.31	2.59	*u.d.l.*
21	*u.d.l.*	2.35	2.08	*u.d.l.*
25	*u.d.l.*	u.d.l.	*u.d.l.*	*u.d.l.*
26	*u.d.l.*	2.06	4.21	4.66
32	*u.d.l.*	1.42	4.75	*u.d.l.*
34	*u.d.l.*	u.d.l.	*u.d.l.*	*u.d.l.*
37	1.58	4.75	5.12	4.2
39	*u.d.l.*	u.d.l.	*u.d.l.*	*u.d.l.*
41	*u.d.l.*	3.00	*u.d.l.*	*u.d.l.*
43	*u.d.l.*	2.17	*u.d.l.*	*u.d.l.*
44	*u.d.l.*	1.88	2.38	*u.d.l.*
53	*u.d.l.*	1.62	*u.d.l.*	*u.d.l.*
55	*u.d.l.*	1.87	1.93	*u.d.l.*
56	*u.d.l.*	u.d.l.	*u.d.l.*	*u.d.l.*

*u.d.l*.: under detection limit (<1.3 Log_10_ cells/ng).

**Table 3:** Statistical analysis of alpha-diversity metrics in dental plaque across study groups and timepoints. Pairwise comparisons were performed using Mann–Whitney U tests (MWU) on deltas (between-group, Control vs. Intervention) and Wilcoxon signed-rank tests (within-group, paired across timepoints). The number of subjects per group (*n*), raw *p*-values, and false discovery rate (FDR)-adjusted q-values are reported (Benjamini–Hochberg correction applied across all tests). Significant results at *p* < 0.05 are highlighted in bold in the text, although none survived FDR correction (q < 0.05).

Variable	Comparison	Test	*n* Control	*n* Probiotic	*p*_Value	*n*	q_Value FDR_BH
observed_features	T_0_–T_2_	MWU (between-group deltas)	24	22	0.3170		0.7411
observed_features	T_0_–T_2_	Wilcoxon (within Control)			0.2522	24	0.7411
observed_features	T_0_–T_2_	Wilcoxon (within PROBIOTIC)			0.6789	22	0.9397
observed_features	T_0_–T_3_	MWU (between-group deltas)	24	22	0.1320		0.5421
observed_features	T_0_–T_3_	Wilcoxon (within Control)			0.3596	24	0.7411
observed_features	T_0_–T_3_	Wilcoxon (within PROBIOTIC)			0.2756	22	0.7411
observed_features	T_2_–T_3_	MWU (between-group deltas)	24	22	0.8604		0.9441
observed_features	T_2_–T_3_	Wilcoxon (within Control)			0.7048	24	0.9397
observed_features	T_2_–T_3_	Wilcoxon (within PROBIOTIC)			0.5661	22	0.8860
faith_pd	T_0_–T_2_	MWU (between-group deltas)	24	22	0.0027		0.0967
faith_pd	T_0_–T_2_	Wilcoxon (within Control)			0.0340	24	0.2447
faith_pd	T_0_–T_2_	Wilcoxon (within PROBIOTIC)			0.0275	22	0.2447
faith_pd	T_0_–T_3_	MWU (between-group deltas)	24	22	0.0969		0.5421
faith_pd	T_0_–T_3_	Wilcoxon (within Control)			0.1355	24	0.5421
faith_pd	T_0_–T_3_	Wilcoxon (within PROBIOTIC)			0.4245	22	0.7457
faith_pd	T_2_–T_3_	MWU (between-group deltas)	24	22	0.4885		0.7994
faith_pd	T_2_–T_3_	Wilcoxon (within Control)			0.8553	24	0.9441
faith_pd	T_2_–T_3_	Wilcoxon (within PROBIOTIC)			0.3705	22	0.7411
pielou_evenness	T_0_–T_2_	MWU (between-group deltas)	24	22	0.0160		0.1925
pielou_evenness	T_0_–T_2_	Wilcoxon (within Control)			0.9441	24	0.9441
pielou_evenness	T_0_–T_2_	Wilcoxon (within PROBIOTIC)			0.0103	22	0.1858
pielou_evenness	T_0_–T_3_	MWU (between-group deltas)	24	22	0.6208		0.8939
pielou_evenness	T_0_–T_3_	Wilcoxon (within Control)			0.9218	24	0.9441
pielou_evenness	T_0_–T_3_	Wilcoxon (within PROBIOTIC)			0.3369	22	0.7411
pielou_evenness	T_2_–T_3_	MWU (between-group deltas)	24	22	0.1834		0.6119
pielou_evenness	T_2_–T_3_	Wilcoxon (within Control)			0.6033	24	0.8939
pielou_evenness	T_2_–T_3_	Wilcoxon (within PROBIOTIC)			0.1207	22	0.5421
shannon_entropy	T_0_–T_2_	MWU (between-group deltas)	24	22	0.4350		0.7457
shannon_entropy	T_0_–T_2_	Wilcoxon (within Control)			0.8553	24	0.9441
shannon_entropy	T_0_–T_2_	Wilcoxon (within PROBIOTIC)			0.1870	22	0.6119
shannon_entropy	T_0_–T_3_	MWU (between-group deltas)	24	22	0.9212		0.9441
shannon_entropy	T_0_–T_3_	Wilcoxon (within Control)			0.7683	24	0.9441
shannon_entropy	T_0_–T_3_	Wilcoxon (within PROBIOTIC)			0.7502	22	0.9441
shannon_entropy	T_2_–T_3_	MWU (between-group deltas)	24	22	0.4222		0.7457
shannon_entropy	T_2_–T_3_	Wilcoxon (within Control)			0.8996	24	0.9441
shannon_entropy	T_2_–T_3_	Wilcoxon (within PROBIOTIC)			0.3535	22	0.7411

MWU stands for Mann–Whitney U test (also known as the Wilcoxon rank-sum test). In practice, applying MWU to the deltas is the non-parametric equivalent of a Time × Treatment interaction: if the deltas differ between groups, this indicates a treatment effect on the temporal trajectory.

**Table 4:** Pairwise comparisons of beta-diversity principal coordinates among time points (T_0_, T_2_, T_3_) in the Control and Intervention groups. *p*-values were obtained using the non-parametric Mann–Whitney U test applied to the first two principal coordinates (PC1, PC2) derived from weighted UniFrac (wUniFrac), unweighted UniFrac (uwUniFrac), Jaccard, and Bray–Curtis dissimilarities. No comparison reached statistical significance (*p* < 0.05), although borderline values were observed for uwUniFrac (Control, T_0_ vs. T_3_, PC1) and for Bray–Curtis (Intervention, T_0_ vs. T_2_, PC2).

Method	Treatment	Comparison	PC1_*p*Value	PC2_*p*Value
wUniFrac	Control	T_0_ vs. T_2_	0.792	0.930
wUniFrac	Control	T_0_ vs. T_3_	0.629	0.660
wUniFrac	Control	T_2_ vs. T_3_	0.982	0.709
wUniFrac	Probiotic	T_0_ vs. T_2_	0.669	0.258
wUniFrac	Probiotic	T_0_ vs. T_3_	0.920	0.900
wUniFrac	Probiotic	T_2_ vs. T_3_	0.763	0.131
uwUniFrac	Control	T_0_ vs. T_2_	0.263	0.895
uwUniFrac	Control	T_0_ vs. T_3_	0.075	0.913
uwUniFrac	Control	T_2_ vs. T_3_	0.895	0.843
uwUniFrac	Probiotic	T_0_ vs. T_2_	0.125	0.960
uwUniFrac	Probiotic	T_0_ vs. T_3_	0.782	0.580
uwUniFrac	Probiotic	T_2_ vs. T_3_	0.258	0.960
Jaccard	Control	T_0_ vs. T_2_	0.913	0.568
Jaccard	Control	T_0_ vs. T_3_	1.000	0.826
Jaccard	Control	T_2_ vs. T_3_	0.913	0.660
Jaccard	Probiotic	T_0_ vs. T_2_	0.513	0.314
Jaccard	Probiotic	T_0_ vs. T_3_	1.000	0.191
Jaccard	Probiotic	T_2_ vs. T_3_	0.529	0.725
bray_curtis	Control	T_0_ vs. T_2_	0.809	1.000
bray_curtis	Control	T_0_ vs. T_3_	0.583	0.895
bray_curtis	Control	T_2_ vs. T_3_	0.758	0.982
bray_curtis	Probiotic	T_0_ vs. T_2_	0.880	0.107
bray_curtis	Probiotic	T_0_ vs. T_3_	0.880	0.651
bray_curtis	Probiotic	T_2_ vs. T_3_	0.940	0.191

**Table 5:** ANOSIM results for beta-diversity comparisons among time points (T_0_, T_2_, T_3_) in the Control and Intervention groups. Analyses were conducted on Euclidean distance matrices calculated from the first two principal coordinates (PC1 and PC2) derived from weighted UniFrac (wUniFrac), unweighted UniFrac (uwUniFrac), Jaccard, and Bray–Curtis dissimilarities. The table reports R statistics and permutation-based *p*-values (999 permutations) for global (T_0_ vs. T_2_ vs. T_3_) and pairwise comparisons. All R values were close to zero or negative, indicating no meaningful separation between time points. Borderline trends were observed for Jaccard (Control, global comparison, *p* = 0.050) and Bray–Curtis (Control, global comparison, *p* = 0.075).

Method	Treatment	Comparison	R_Statistic	*p*_Value
wUniFrac	Control	Global (T_0_, T_2_, T_3_)	−0.02659	0.1150
wUniFrac	Control	T_0_ vs. T_2_	−0.01927	0.4240
wUniFrac	Control	T_0_ vs. T_3_	−0.02375	0.3150
wUniFrac	Control	T_2_ vs. T_3_	−0.03729	0.0920
wUniFrac	Probiotic	Global (T_0_, T_2_, T_3_)	−0.01680	0.5070
wUniFrac	Probiotic	T_0_ vs. T_2_	−0.01341	0.6900
wUniFrac	Probiotic	T_0_ vs. T_3_	−0.01885	0.5510
wUniFrac	Probiotic	T_2_ vs. T_3_	−0.01808	0.5900
uwUniFrac	Control	Global (T_0_, T_2_, T_3_)	−0.01155	0.5930
uwUniFrac	Control	T_0_ vs. T_2_	−0.01201	0.6860
uwUniFrac	Control	T_0_ vs. T_3_	0.00774	0.7850
uwUniFrac	Control	T_2_ vs. T_3_	−0.03033	0.2330
uwUniFrac	Probiotic	Global (T_0_, T_2_, T_3_)	−0.00192	0.9510
uwUniFrac	Probiotic	T_0_ vs. T_2_	0.02693	0.4040
uwUniFrac	Probiotic	T_0_ vs. T_3_	−0.02314	0.4790
uwUniFrac	Probiotic	T_2_ vs. T_3_	−0.01070	0.7450
Jaccard	Control	Global (T_0_, T_2_, T_3_)	−0.03642	0.0500
Jaccard	Control	T_0_ vs. T_2_	−0.03035	0.2030
Jaccard	Control	T_0_ vs. T_3_	−0.04006	0.1090
Jaccard	Control	T_2_ vs. T_3_	−0.03862	0.0860
Jaccard	Probiotic	Global (T_0_, T_2_, T_3_)	−0.02243	0.2510
Jaccard	Probiotic	T_0_ vs. T_2_	−0.02718	0.2940
Jaccard	Probiotic	T_0_ vs. T_3_	−0.01016	0.7350
Jaccard	Probiotic	T_2_ vs. T_3_	−0.03012	0.2450
bray_curtis	Control	Global (T_0_, T_2_, T_3_)	−0.03068	0.0750
bray_curtis	Control	T_0_ vs. T_2_	−0.03538	0.1300
bray_curtis	Control	T_0_ vs. T_3_	−0.02676	0.2570
bray_curtis	Control	T_2_ vs. T_3_	−0.03037	0.1530
bray_curtis	Probiotic	Global (T_0_, T_2_, T_3_)	−0.02001	0.3850
bray_curtis	Probiotic	T_0_ vs. T_2_	−0.01228	0.7180
bray_curtis	Probiotic	T_0_ vs. T_3_	−0.02507	0.4020
bray_curtis	Probiotic	T_2_ vs. T_3_	−0.02328	0.4740

**Table 6:** Differentially abundant bacterial taxa between the Intervention and Control groups. (A) Taxa significantly modulated at T_2_ (end of probiotic treatment). (B) Taxa significantly modulated at T_3_ (post-treatment follow-up). Results derive from a DESeq2-like model testing the time × treatment interaction. Coefficients (Coef_log_rate) indicate the relative change in abundance in the Intervention group compared to the Control group at each timepoint; positive values indicate a greater increase (or smaller decrease), negative values indicate the opposite. The table reports taxonomic classification, Coef_log_rate, q-value, and effect direction.

**A**			
**Taxon**	**Coef_log_rate**	**qval**	**Direction**
p_*Actinobacteriota*;c_*Actinomycetes*;o_*Micrococcales*	−2.54	0.0020	↓ in Probiotic
p_*Actinobacteriota*;c_*Actinomycetes*;o_*Micrococcales*;f_*Micrococcaceae*	−2.83	0.0000	↓ in Probiotic
p_*Actinobacteriota*;c_*Actinomycetes*;o_*Micrococcales*;f_*Micrococcaceae*;g_*Rothia*	−1.47	0.0140	↓ in Probiotic
p_*Actinobacteriota;*c_*Actinomycetes*;o_*Propionibacteriales*	−1.24	0.0490	↓ in Probiotic
p_*Bacteroidota*;c_*Bacteroidia*;o_*Bacteroidales*;f_*Rikenellaceae*;g_*Rikenellaceae*_RC9_gut_group	−4.27	0.0010	↓ in Probiotic
p_*Bacteroidota*;c_*Bacteroidia*;o_*Flavobacteriales*	−1.50	0.0040	↓ in Probiotic
p_*Bacteroidota*;c_*Bacteroidia*;o_*Flavobacteriales*;f_*Flavobacteriaceae*	−1.38	0.0020	↓ in Probiotic
p_*Bacteroidota*;c_*Bacteroidi*a;o_*Flavobacteriales*;f_*Flavobacteriaceae*;g_*Capnocytophaga*	−1.47	0.0080	↓ in Probiotic
p_*Bacteroidota*;c_*Bacteroidia*;o_*Flavobacteriales*;f_*Weeksellaceae*	−1.33	0.0310	↓ in Probiotic
p_*Bacteroidota*;c_*Bacteroidia*;o_*Flavobacteriales*;f_*Weeksellaceae*;g_*Bergeyella*	−1.44	0.0010	↓ in Probiotic
p_*Bacteroidota*;c_*Bacteroidia*;o_*Sphingobacteriales*;f_*Lentimicrobiaceae*;g_*Lentimicrobium*	−27.13 ^a^	0.0000	↓ in Probiotic
p_*Bacillota*;c_*Bacilli*;o_*Lactobacillales*	−1.62	0.0080	↓ in Probiotic
p_*Bacillota*;c_*Bacilli*;o_*Lactobacillales*;f_*Carnobacteriaceae*	−1.19	0.0490	↓ in Probiotic
p_*Bacillota*;c_*Bacilli*;o_*Lactobacillales*;f_*Streptococcaceae*	−1.35	0.0370	↓ in Probiotic
p_*Bacillota*;c_*Bacilli*;o_*Staphylococcales*	−1.65	0.0040	↓ in Probiotic
p_*Bacillota*;c_*Bacilli*;o_*Staphylococcales*;f_*Gemellaceae*	−1.48	0.0050	↓ in Probiotic
p_*Bacillota*;c_*Clostridia*;o_*Lachnospirales*;f_*Lachnospiraceae*;g_*Lachnoanaerobaculum*	1.86	0.0220	↑ in Probiotic
p_*Patescibacteria*;c_*Saccharimonadia*;o_*Saccharimonadales*;f_*Saccharimonadaceae*;g_*Candidatus*_*Saccharimonas*	−27.05 ^a^	0.0000	↓ in Probiotic
p_*Pseudomonadota*;c_*Alphaproteobacteria*;o_*Rhizobiales*;f_*Xanthobacteraceae*	−24.64 ^a^	0.0000	↓ in Probiotic
p_*Pseudomonadota*;c_*Gammaproteobacteria*;o_*Burkholderiales*	−1.41	0.0040	↓ in Probiotic
p_*Pseudomonadota*;c_*Gammaproteobacteria*;o_*Burkholderiales*;f_*Burkholderiaceae*	−2.69	0.0000	↓ in Probiotic
p_*Pseudomonadota*;c_*Gammaproteobacteria*;o_*Burkholderiales*;f_*Burkholderiaceae*;g_*Lautropia*	−2.88	0.0000	↓ in Probiotic
p_*Pseudomonadota*;c_*Gammaproteobacteria*;o_*Burkholderiales*;f_*Neisseriaceae*	−1.64	0.0030	↓ in Probiotic
p_*Pseudomonadota*;c_*Gammaproteobacteria*;o_*Burkholderiales*;f_*Neisseriaceae*;g_*Neisseria*	−1.11	0.0350	↓ in Probiotic
p_*Pseudomonadota*;c_*Gammaproteobacteria*;o_*Pasteurellales*	−1.94	0.0000	↓ in Probiotic
p_*Pseudomonadota*;c_*Gammaproteobacteria*;o_*Pasteurellales*;f_*Pasteurellaceae*	−1.84	0.0070	↓ in Probiotic
p_*Pseudomonadota*;c_*Gammaproteobacteria*;o_*Pseudomonadales*;f_*Moraxellaceae* ^b^	−4.61	0.0050	↓ in Probiotic
**B**			
**Taxon**	**Coef_log_rate**	**qval**	**Direction**
p_*Actinobacteriota*;c_*Actinomycetes*;o_*Actinomycetales*;f_*Actinomycetaceae*;g_*Actinomyces*	−2.13	0.0050	↓ in Probiotic
p_*Actinobacteriota*;c_*Actinomycetes*;o_*Actinomycetales*;f_*Actinomycetaceae*;g_F0332	3.80	0.0120	↑ in Probiotic
p_*Bacteroidota*;c_*Bacteroidia*;o_*Bacteroidales*;f_*Prevotellaceae*;g_*Alloprevotella*	−1.21	0.0230	↓ in Probiotic
p_*Bacteroidota*;c_*Bacteroidia*;o_*Bacteroidales*;f_*Prevotellaceae*;g_*Prevotella*	−2.96	0.0220	↓ in Probiotic
p_*Bacteroidota*;c_*Bacteroidia*;o_*Bacteroidales*;f_*Rikenellaceae*;g_*Rikenellaceae*_RC9_gut_group	−24.78 ^a^	0.0000	↓ in Probiotic
p_*Bacteroidota*;c_*Bacteroidia*;o_*Bacteroidales*;f_*Tannerellaceae*;g_*Tannerella*	−25.91 ^a^	0.0000	↓ in Probiotic
p_*Bacillota*;c_*Bacilli*;o_*Staphylococcales*;f_*Gemellaceae*;g_*Gemella*	−1.32	0.0370	↓ in Probiotic
p_*Bacillota*;c_*Clostridia*;o_*Lachnospirales*;f_*Lachnospiraceae*	−1.43	0.0050	↓ in Probiotic
p_*Bacillota*;c_*Negativicutes*;o_*Veillonellales-Selenomonadales*;f_*Selenomonadaceae*	2.10	0.0030	↑ in Probiotic
p_*Bacillota*;c_*Negativicutes*;o_*Veillonellales-Selenomonadales*;f_*Selenomonadaceae*;g_*Selenomonas*	−1.42	0.0350	↓ in Probiotic
p_*Fusobacteriota*;c_*Fusobacteriia*;o_*Fusobacteriales*;f_*Leptotrichiaceae*;g_*Leptotrichia*	−2.89	0.0000	↓ in Probiotic
p_*Patescibacteria*;c_*Gracilibacteria*;o_*Absconditabacteriales*_(SR1)	−1.38	0.0230	↓ in Probiotic
p_*Patescibacteria*;c_*Saccharimonadia*;o_*Saccharimonadales*;f_*Saccharimonadaceae*;g_*Candidatus*_*Saccharimonas*	−27.99 ^a^	0.0000	↓ in Probiotic
p_*Pseudomonadota*;c_*Gammaproteobacteria*;o_*Burkholderiales*;f_*Neisseriaceae*	−1.20	0.0310	↓ in Probiotic
p_*Pseudomonadota*;c_*Gammaproteobacteria*;o_*Burkholderiales*;f_*Neisseriaceae*;g_*Kingella*	−1.52	0.0340	↓ in Probiotic
p_*Pseudomonadota*;c_*Gammaproteobacteria*;o_*Pseudomonadales*	−6.34	0.0000	↓ in Probiotic
p_*Pseudomonadota*;c_*Gammaproteobacteria*;o_*Pseudomonadales*;f_*Moraxellaceae* ^b^	−5.23	0.0000	↓ in Probiotic
p_*Pseudomonadota*;c_*Gammaproteobacteria*;o_*Pseudomonadales*;f_*Moraxellaceae*;g_*Moraxella* ^b^	−7.15	0.0000	↓ in Probiotic
p_*Spirochaetota*;c_*Spirochaetia*;o_*Spirochaetales*;f_*Spirochaetaceae*;g_*Treponema*	−26.11 ^a^	0.0000	↓ in Probiotic

^a^, these extreme Coef_log_rate values largely reflect the very low prevalence of such sparse taxa across samples and should therefore be interpreted with caution; ^b^, taxa detected in less than 25% of the samples; p: phylum; c: class; o: order; f: family; g: genus. The arrows indicate whether the reported taxa increase (↑) or decrease (↓) in Probiotic group.

## Data Availability

The data supporting the findings of this study are publicly available in the University of Milan Dataverse repository at the following DOI: https://doi.org/10.13130/RD_UNIMI/YF2NZV. All datasets generated and analyzed during the current study have been deposited and can be accessed.

## Supplementary Materials

The following supporting information can be downloaded at: https://www.mdpi.com/article/10.3390/nu18060904/s1, File S1. CONSORT 2010 checklist of information to include when reporting a randomized trial, File S2. The chewing gum production process, File S3. Schematic representation of the adopted bioinformatic workflow for 16S rRNA gene amplicon sequencing analysis, File S4. Participant-level data from a clinical trial, detailing demographics, group allocation, protocol adherence, and reported adverse effects across multiple time points.

## Abbreviations

The following abbreviations are used in this manuscript:

CFU	Colony-Forming Units
MRD	Maximum Recovery Diluent
ppm	Parts Per Million
qPCR	Quantitative Polymerase Chain Reaction,
DNA	Deoxyribonucleic Acid
rRNA	Ribosomal Ribonucleic Acid
u.d.l.	Under the Detection Limit
MWU	Mann–Whitney U
FDR	False Discovery Rate
NO	Nitrate Oxidase
