# Supplementary material for: Assessing the Impact of Heyndrickxia coagulans Administered Through Sugar-Free Chewing Gum on Dental Biofilm: A Double-Blind Randomized Controlled Trial
Source: Nutrients. 2026 Mar 12;18(6):904. doi: 10.3390/nu18060904 (PMC13029583; doi:10.3390/nu18060904)
Supplement: Supplementary file 1 [file nutrients-18-00904-s001.zip › nutrients-4173205-supplementary.pdf]

Assessing the Impact on dental biofilm of *H. coagulans* administered through sugar-free chewing gum: a double-blind randomized controlled trial

Supplementary file

File S1. CONSORT 2010 checklist of information to include when reporting a randomized trial.

File S2. The chewing gum production process.

File S3. Schematic representation of the adopted bioinformatic workflow for 16S rRNA gene amplicon sequencing analysis.

File S4. Participant-level data from a clinical trial, detailing demographics, group allocation, protocol adherence, and reported adverse effects across multiple time points.

File S1. CONSORT 2010 checklist of information to include when reporting a randomized trial.

Section/Topic	Item No	Checklist item	Reported on page No
**Title and abstract**			
	1a	Identification as a randomised trial in the title	1
	1b	Structured summary of trial design, methods, results, and conclusions (for specific guidance see CONSORT for abstracts)	1
**Introduction**			
Background and objectives	2a	Scientific background and explanation of rationale	2
	2b	Specific objectives or hypotheses	2
**Methods**			
Trial design	3a	Description of trial design (such as parallel, factorial) including allocation ratio	2-3
	3b	Important changes to methods after trial commencement (such as eligibility criteria), with reasons	n.a.
Participants	4a	Eligibility criteria for participants	3
	4b	Settings and locations where the data were collected	3
Interventions	5	The interventions for each group with sufficient details to allow replication, including how and when they were actually administered	3-5
Outcomes	6a	Completely defined pre-specified primary and secondary outcome measures, including how and when they were assessed	5-6
	6b	Any changes to trial outcomes after the trial commenced, with reasons	n.a.
Sample size	7a	How sample size was determined	3
	7b	When applicable, explanation of any interim analyses and stopping guidelines	n.a.
Randomisation:			
Sequence generation	8a	Method used to generate the random allocation sequence	3
	8b	Type of randomisation; details of any restriction (such as blocking and block size)	3

Allocation concealment mechanism	9	Mechanism used to implement the random allocation sequence (such as sequentially numbered containers), describing any steps taken to conceal the sequence until interventions were assigned	3
Implementation	10	Who generated the random allocation sequence, who enrolled participants, and who assigned participants to interventions	3
Blinding	11a	If done, who was blinded after assignment to interventions (for example, participants, care providers, those assessing outcomes) and how	3
	11b	If relevant, description of the similarity of interventions	n.a.
Statistical methods	12a	Statistical methods used to compare groups for primary and secondary outcomes	6-7
	12b	Methods for additional analyses, such as subgroup analyses and adjusted analyses	6-7
**Results**			
Participant flow (a diagram is strongly recommended)	13a	For each group, the numbers of participants who were randomly assigned, received intended treatment, and were analysed for the primary outcome	7-8
	13b	For each group, losses and exclusions after randomisation, together with reasons	7-8
Recruitment	14a	Dates defining the periods of recruitment and follow-up	7-8
	14b	Why the trial ended or was stopped	7-8
Baseline data	15	A table showing baseline demographic and clinical characteristics for each group	8-9
Numbers analysed	16	For each group, number of participants (denominator) included in each analysis and whether the analysis was by original assigned groups	8-9
Outcomes and estimation	17a	For each primary and secondary outcome, results for each group, and the estimated effect size and its precision (such as 95% confidence interval)	9-18
	17b	For binary outcomes, presentation of both absolute and relative effect sizes is recommended	9-18
Ancillary analyses	18	Results of any other analyses performed, including subgroup analyses and adjusted analyses, distinguishing pre-specified from exploratory	9-18

Harms	19	All important harms or unintended effects in each group (for specific guidance see CONSORT for harms)	8-9
**Discussion**			
Limitations	20	Trial limitations, addressing sources of potential bias, imprecision, and, if relevant, multiplicity of analyses	16
Generalisability	21	Generalisability (external validity, applicability) of the trial findings	14-16
Interpretation	22	Interpretation consistent with results, balancing benefits and harms, and considering other relevant evidence	14-16
**Other information**			
Registration	23	Registration number and name of trial registry	2-3
Protocol	24	Where the full trial protocol can be accessed, if available	2-3
Funding	25	Sources of funding and other support (such as supply of drugs), role of funders	17

File S2. The chewing gum production process.

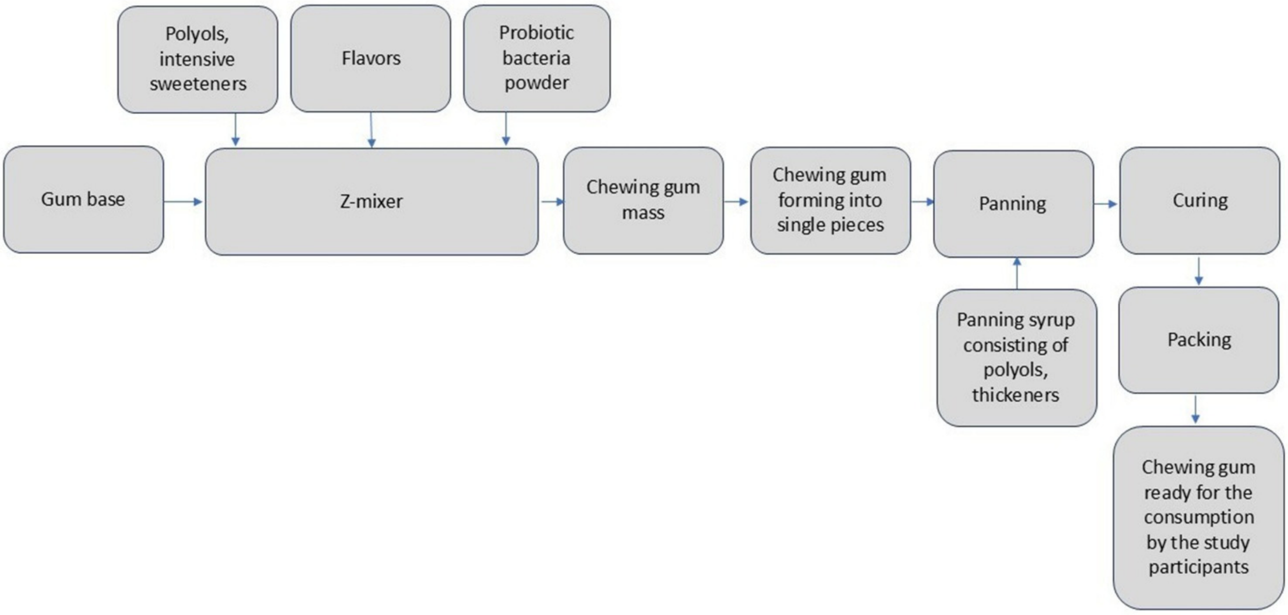

File S3. Schematic representation of the adopted bioinformatic workflow for 16S rRNA gene amplicon sequencing analysis. Raw paired-end FASTQ® files were processed in QIIME 2™ (version 2024.5, q2-amplicon distribution. Caporaso Lab, Northern Arizona University, Flagstaff, AZ, USA). After demultiplexing, reads were quality-filtered, denoised, merged, and chimera-checked with the DADA2® plugin, generating an amplicon sequence variant (ASV) feature table and representative sequences. Alpha- and beta 6 diversity metrics were computed from the ASV table using the q2-diversity plugin. Specifically, within-sample diversity was assessed by observed features (richness), Faith's phylogenetic diversity (Faith's PD), Pielou's evenness, and Shannon entropy. Between-sample diversity was evaluated using four dissimilarity metrics: weighted UniFrac, unweighted UniFrac, Jaccard, and Bray–Curtis. Taxonomic classification of ASVs was performed with a Naive Bayes classifier trained on SILVA release 138 (99% OTUs, 515F–806R region). Abundances were summarized using taxa bar plots and exported at multiple taxonomic levels (from ASV to phylum). Inferential differential-abundance analysis was conducted with DESeq2 (Bioconductor) on raw counts aggregated by rank (phylum, class, order, family, genus), considering taxa present in ≥25% of samples (see paragraph describing statistics for more details).

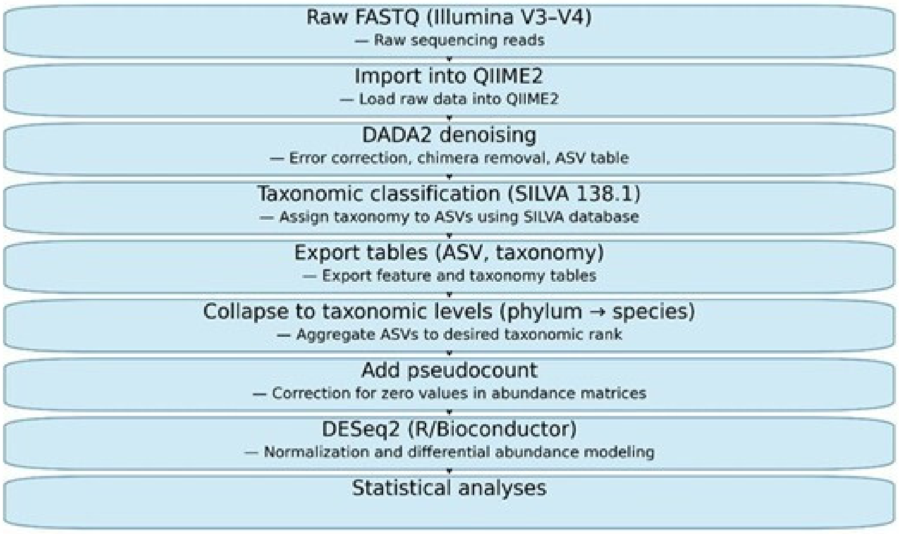

File S4. Participant-level data from a clinical trial, detailing demographics, group allocation, protocol adherence, and reported adverse effects across multiple time points.

ID	Age (years)	Gender	Group	START WASH-OUT	T_0	Compliance (number of chewing gum skipped)	Unpleasant flavour, texture, and size of chewing gum	Gastro-intestinal disorders (pain/ bloating/ constipation/ reflux)	T_1	Compliance (number of chewing gum skipped)	Unpleasant flavour, texture, and size of chewing gum	Gastro-intestinal disorders (pain/ bloating/ constipation/ reflux)	T_2	T_3
1	50	F	Placebo	x	x	3	x	x	x				x	x
2	24	F	Placebo	x	x				x				x	x
3	48	M	Intervention	x	x		x		x				x	x
4	26	F	Intervention	x	x			x	x		x		x	x
5	37	F	Intervention	x	x	2		x	x	10			x	x
6	53	M	Placebo	x	x				x			x	x	x
7	39	F	Placebo	x	x			x	x			x	x	x
8	29	M	Intervention	x	x		x		x	1	x	x	x	x
9	51	F	Intervention	x	x			x	x				x	x
10	30	F	Intervention	x	x		x		x			x	x	x
11	Refused enrolment owing to challenges in ensuring adherence to the study protocol													
12	27	F	Intervention	x	x			x	x				x	x
13	26	F	Placebo	x	x		x		x				x	x
14	28	F	Placebo	x	x	x			Excluded (dislike of the chewing gum flavour)					
15	27	F	Placebo	x	x	2	x	x	x	3		x	x	x
16	Excluded due to a potential allergy to the chewing gum components													

17	24	F	Placebo	x	x				x				x	x
18	23	M	Placebo	x	x				x				x	x
19	24	F	Placebo	x	x				x				x	x
20	23	F	Intervention	x	x	2			x				x	x
21	22	F	Intervention	x	x				x	4			x	x
22	26	f	Intervention	x	Excluded due to non-adherence during the wash-out period									
23	28	F	Intervention	x	x			x	Excluded (g-i disorders)					
24	27	F	Placebo	x	x	4			x	4			x	x
25	29	F	Intervention	x	x				x	4			x	x
26	55	F	Intervention	x	x	2			x				x	x
27	28	F	Placebo	x	x				x				x	x
28	24	F	Placebo	x	x				x				x	x
29	29	F	Placebo	x	x				x				x	x
30	21	M	Placebo	x	x				x				x	x
31	20	M	Placebo	x	x	5			x				x	x
32	21	F	Intervention	x	x				x	5			x	x
33	Refused enrolment owing to challenges in ensuring adherence to the study protocol													
34	22	F	Intervention	x	x			x	x		x	x	x	x
35	Excluded due to a potential allergy to the chewing gum components													
36	25	F	Placebo	x	x				x	Excluded (missed follow-up)				
37	21	F	Intervention	x	x				x				x	x
38	20	F	Placebo	x	x				x				x	x

39	21	F	Intervention	x	x				x				x	x
40	20	F	Placebo	x	x				x			x	Excluded (g-i disorders)	
41	20	F	Intervention	x	x				x				x	x
42	22	F	Placebo	x	x				x				x	x
43	21	F	Intervention	x	x				x				x	x
44	45	F	Intervention	x	x			x	x			x	x	x
45	29	F	Placebo	x	x				x				x	x
46	23	F	Placebo	x	x				x				x	x
47	26	F	Placebo	x	x			x	x				x	x
48	24	F	Intervention	Excluded owing to health complications that emerged after enrolment										
49	24	F	Placebo	x	x			x	x				x	x
50	27	M	Intervention	x	x			x	Excluded (g-i disorders)					
51	23	F	Placebo	x	x				x				x	x
52	24	F	Placebo	x	x				x				x	x
53	23	F	Intervention	x	x				x				x	x
54	24	F	Intervention	Excluded owing to health complications that emerged after enrolment										
55	24	F	Intervention	x	x				x				x	x
56	23	F	Intervention	x	x				x				x	x

N: number; SD: standard deviation; M: male; F: female; g-i: gastro-intestinal.
